# 3-Ethyl-4-[(*E*)-2-methyl­benzyl­idene­amino]-1*H*-1,2,4-triazole-5(4*H*)-thione

**DOI:** 10.1107/S1600536808019867

**Published:** 2008-07-05

**Authors:** Shan-Heng Wang, Ying-Li Xu, Pei-Jin Xie, Wen-Long Wang, Shang Shan

**Affiliations:** a College of Chemical Engineering and Materials Science, Zhejiang University of Technology, People’s Republic of China

## Abstract

Crystals of the title compound, C_12_H_14_N_4_S, were obtained from a condensation reaction of 4-amino-3-ethyl-1*H*-1,2,4-triazole-5(4*H*)-thione and 2-methyl­benzaldehyde. In the mol­ecular structure, there is a short N=C double bond [1.255 (2) Å], and the benzene and triazole rings are located on opposite sites of this double bond. The two rings are approximately parallel to each other, the dihedral angle being 1.75 (11)°. A partially overlapped arrangement is observed between the nearly parallel triazole and benzene rings of adjacent mol­ecules; the perpendicular distance of the centroid of the triazole ring from the benzene ring is 3.482 Å, indicating the existence of π–π stacking in the crystal structure.

## Related literature

For general background, see: Okabe *et al.* (1993 ▶); Shan *et al.* (2003 ▶). For related structures, see: Fan *et al.* (2008 ▶); Shan *et al.* (2004 ▶, 2008 ▶). For the thickness of the aromatic ring, see: Cotton & Wilkinson (1972 ▶).
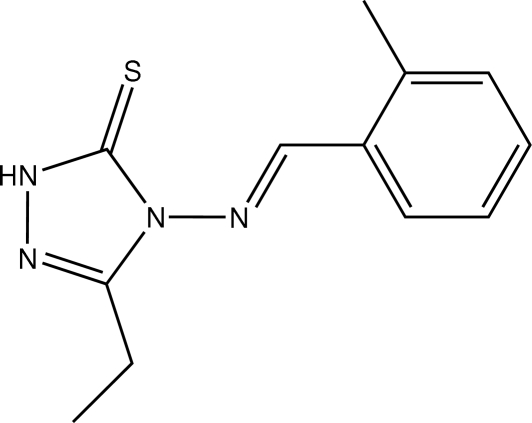


## Experimental

### 

#### Crystal data

C_12_H_14_N_4_S
*M* *_r_* = 246.33
Monoclinic, 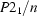

*a* = 7.7255 (15) Å
*b* = 15.411 (3) Å
*c* = 10.685 (2) Å
β = 101.032 (12)°
*V* = 1248.7 (4) Å^3^
*Z* = 4
Mo *K*α radiation
μ = 0.24 mm^−1^
*T* = 295 (2) K
0.32 × 0.28 × 0.24 mm

#### Data collection

Rigaku R-AXIS RAPID IP diffractometer
Absorption correction: none
12354 measured reflections
2860 independent reflections
1777 reflections with *I* > 2σ(*I*)
*R*_int_ = 0.061

#### Refinement

*R*[*F* ^2^ > 2σ(*F* ^2^)] = 0.047
*wR*(*F* ^2^) = 0.120
*S* = 1.03
2860 reflections
156 parameters
H-atom parameters constrained
Δρ_max_ = 0.18 e Å^−3^
Δρ_min_ = −0.21 e Å^−3^

### 

Data collection: *PROCESS-AUTO* (Rigaku, 1998 ▶); cell refinement: *PROCESS-AUTO*; data reduction: *CrystalStructure* (Rigaku/MSC, 2002 ▶); program(s) used to solve structure: *SIR92* (Altomare *et al.*, 1993 ▶); program(s) used to refine structure: *SHELXL97* (Sheldrick, 2008 ▶); molecular graphics: *ORTEP-3 for Windows* (Farrugia, 1997 ▶); software used to prepare material for publication: *WinGX* (Farrugia, 1999 ▶).

## Supplementary Material

Crystal structure: contains datablocks I, global. DOI: 10.1107/S1600536808019867/xu2434sup1.cif

Structure factors: contains datablocks I. DOI: 10.1107/S1600536808019867/xu2434Isup2.hkl

Additional supplementary materials:  crystallographic information; 3D view; checkCIF report

## Figures and Tables

**Table 1:** Hydrogen-bond geometry (Å, °)

*D*—H⋯*A*	*D*—H	H⋯*A*	*D*⋯*A*	*D*—H⋯*A*
N1—H1N⋯S^i^	0.93	2.37	3.2899 (19)	169
C5—H5⋯S	0.93	2.54	3.239 (2)	132

Symmetry code: (i) 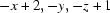.

**Fig. 1.**
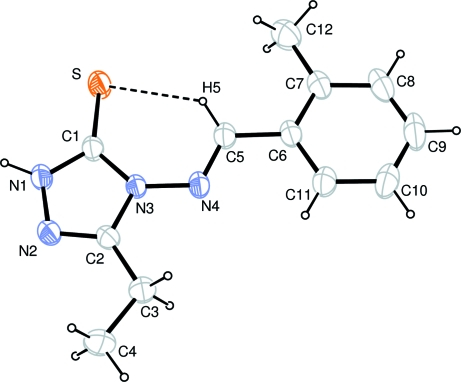
The molecular structure of the title compound with 40% probability displacement ellipsoids for non-H atoms. Dashed line indicates hydrogen bonding.

**Fig. 2.**
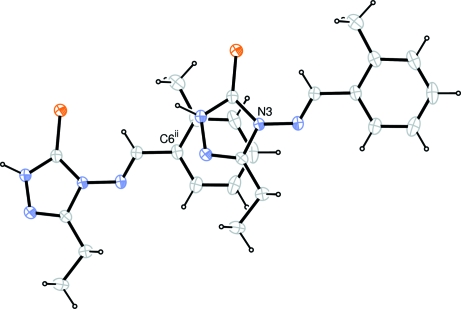
A diagram showing π-π stacking between aromatic rings [symmetry code: (ii) 1 + x, y, z].

## supplementary crystallographic information

### Comment

Since some hydrazone derivatives have shown to be potential DNA damaging and mutagenic agents (Okabe *et al.*, 1993), a series of new hydrazone derivatives have been prepared in our laboratory (Shan *et al.*, 2003). As part of the ongoing investigation, the title compound has recently been prepared and its crystal structure is reported here.

The molecular structure of the title compound is shown in Fig. 1. The N4—C5 bond distance of 1.255 (2) Å is significantly shorter than C═N bond distances found in related hydrazone structures, i.g. 1.295 (2) Å in (*E*)-3-methoxyacetophenone 4-nitrophenylhydrazone (Fan *et al.*, 2008), 1.2977 (18) Å in (E)-2-furyl methyl ketone 2,4-dinitrophenylhydrazone (Shan *et al.*, 2008) and 1.293 (2) Å in benzylideneacetone 2,4-dinitrophenylhydrazone (Shan *et al.*, 2004). The benzene and triazole rings are located on the opposite sites of the N4═C5 double bond, the molecule assumes an E-configuration.

The molecule displays a nearly coplanar structure except for methyl H atoms, the maximum atomic deviation for non-H atom is 0.1457 (18) Å (N4), and the atomic deviations for ehtyl C3 and C4 atoms are 0.011 (2) and 0.037 (2) Å, respectively. The methine group is linked to the triazolethione *via* intramolecular C5—H5···S hydrogen bonding (Fig. 1 and Table 1). The adjacent molecules are linked together with N1—H1N···S hydrogen bonding (Table 1), forming the centro-symmetric supramolecular dimer.

A partially overlapped arrangement is observed between the nearly parallel triazole ring and benzene ring of the adjacent molecule [dihedral angle 1.75 (11)°] (Fig. 2), the perpendicular distance of the centroid of the N3-triazole ring on the C6^ii^-benzene ring is 3.482 Å and the perpendicular distance of the centroid of the C6^ii^-benzene ring on the N3-triazole ring is 3.504 Å [symmetry code: (ii) 1 + *x*, *y*, *z*], these are significantly shorter than the van der Waals thickness of the aromatic ring (3.7 Å; Cotton & Wilkinson, 1972) and suggest the existence of π-π stacking in the crystal structure.

### Experimental

4-Amino-3-ethyl-1*H*-1,2,4-triazole-5(4*H*)-thione (0.29 g, 2 mmol) was dissolved in ethanol (25 ml), then acetic acid (1 ml) was added slowly to the ethanol solution with stirring. The solution was heated at 333 K for several minutes until the solution cleared. 2-Methylbenzaldehyde (0.24 g, 2 mmol) was then dropped slowly into the solution, and the mixture was refluxed for 5 h. After the solution had cooled to room temperature yellow powder crystals appeared. The powder crystals were separated and washed with water three times. Single crystals of the title compound were obtained by recrystallization from an absolute ethanol solution.

### Refinement

H atom bonded to N atom was located in a difference Fourier map and refined as riding in its as-found relative position with *U*_iso_(H) = 1.5*U*_eq_(N). Methyl H atoms were placed in calculated positions with C—H = 0.96 Å and the torsion angles were refined to fit the electron density, *U*_iso_(H) = 1.5*U*_eq_(C). Other H atoms were placed in calculated positions with C—H = 0.93 (aromatic) and 0.97 Å (methylene), and refined in riding mode with *U*_iso_(H) = 1.2*U*_eq_(C).

### Crystal data

**Table:**

C_12_H_14_N_4_S	*F*_000_ = 520
*M**_r_* = 246.33	*D*_x_ = 1.310 Mg m^−^^3^
Monoclinic, *P*2_1_/*n*	Mo *K*α radiation λ = 0.71073 Å
Hall symbol: -P 2yn	Cell parameters from 4278 reflections
*a* = 7.7255 (15) Å	θ = 2.8–24.5º
*b* = 15.411 (3) Å	µ = 0.24 mm^−^^1^
*c* = 10.685 (2) Å	*T* = 295 (2) K
β = 101.032 (12)º	Prism, yellow
*V* = 1248.7 (4) Å^3^	0.32 × 0.28 × 0.24 mm
*Z* = 4	

### Data collection

**Table:**

Rigaku R-AXIS RAPID IP diffractometer	2860 independent reflections
Radiation source: fine-focus sealed tube	1777 reflections with *I* > 2σ(*I*)
Monochromator: graphite	*R*_int_ = 0.061
Detector resolution: 10.00 pixels mm^-1^	θ_max_ = 27.6º
*T* = 295(2) K	θ_min_ = 2.6º
ω scans	*h* = −9→10
Absorption correction: none	*k* = −20→16
12354 measured reflections	*l* = −13→13

### Refinement

**Table:**

Refinement on *F*^2^	Secondary atom site location: difference Fourier map
Least-squares matrix: full	Hydrogen site location: inferred from neighbouring sites
*R*[*F*^2^ > 2σ(*F*^2^)] = 0.047	H-atom parameters constrained
*wR*(*F*^2^) = 0.121	*w* = 1/[σ^2^(*F*_o_^2^) + (0.0463*P*)^2^ + 0.2633*P*] where *P* = (*F*_o_^2^ + 2*F*_c_^2^)/3
*S* = 1.03	(Δ/σ)_max_ < 0.001
2860 reflections	Δρ_max_ = 0.18 e Å^−^^3^
156 parameters	Δρ_min_ = −0.21 e Å^−^^3^
Primary atom site location: structure-invariant direct methods	Extinction correction: none

### Special details

**Table:**

Geometry. All e.s.d.'s (except the e.s.d. in the dihedral angle between two l.s. planes) are estimated using the full covariance matrix. The cell e.s.d.'s are taken into account individually in the estimation of e.s.d.'s in distances, angles and torsion angles; correlations between e.s.d.'s in cell parameters are only used when they are defined by crystal symmetry. An approximate (isotropic) treatment of cell e.s.d.'s is used for estimating e.s.d.'s involving l.s. planes.
Refinement. Refinement of *F*^2^ against ALL reflections. The weighted *R*-factor *wR* and goodness of fit *S* are based on *F*^2^, conventional *R*-factors *R* are based on *F*, with *F* set to zero for negative *F*^2^. The threshold expression of *F*^2^ > σ(*F*^2^) is used only for calculating *R*-factors(gt) *etc*. and is not relevant to the choice of reflections for refinement. *R*-factors based on *F*^2^ are statistically about twice as large as those based on *F*, and *R*- factors based on ALL data will be even larger.

### Fractional atomic coordinates and isotropic or equivalent isotropic displacement parameters (Å^2^)

**Table:**

	*x*	*y*	*z*	*U*_iso_*/*U*_eq_	
S	0.70986 (7)	−0.00644 (3)	0.39484 (6)	0.0542 (2)	
N1	0.9437 (2)	0.12184 (11)	0.47565 (18)	0.0453 (5)	
H1N	1.0320	0.0826	0.5095	0.068*	
N2	0.9697 (2)	0.20996 (11)	0.47450 (18)	0.0449 (5)	
N3	0.70093 (19)	0.17368 (10)	0.37970 (15)	0.0356 (4)	
N4	0.5343 (2)	0.19429 (11)	0.31007 (17)	0.0424 (5)	
C1	0.7829 (3)	0.09601 (13)	0.4175 (2)	0.0388 (5)	
C2	0.8200 (3)	0.24045 (13)	0.4152 (2)	0.0387 (5)	
C3	0.7750 (3)	0.33278 (13)	0.3886 (2)	0.0486 (6)	
H3A	0.6760	0.3482	0.4279	0.058*	
H3B	0.7391	0.3408	0.2973	0.058*	
C4	0.9288 (3)	0.39305 (14)	0.4383 (3)	0.0627 (7)	
H4A	0.9624	0.3868	0.5291	0.094*	
H4B	0.8942	0.4520	0.4180	0.094*	
H4C	1.0270	0.3784	0.3991	0.094*	
C5	0.4118 (3)	0.13985 (14)	0.3022 (2)	0.0443 (6)	
H5	0.4330	0.0856	0.3402	0.053*	
C6	0.2346 (3)	0.16241 (14)	0.2325 (2)	0.0412 (5)	
C7	0.1079 (3)	0.09783 (15)	0.1958 (2)	0.0484 (6)	
C8	−0.0589 (3)	0.1238 (2)	0.1313 (2)	0.0631 (7)	
H8	−0.1454	0.0820	0.1064	0.076*	
C9	−0.0980 (3)	0.2091 (2)	0.1040 (3)	0.0688 (8)	
H9	−0.2105	0.2245	0.0618	0.083*	
C10	0.0279 (3)	0.27235 (18)	0.1386 (2)	0.0621 (7)	
H10	0.0019	0.3302	0.1182	0.074*	
C11	0.1935 (3)	0.24901 (15)	0.2038 (2)	0.0502 (6)	
H11	0.2783	0.2916	0.2288	0.060*	
C12	0.1452 (3)	0.00349 (16)	0.2212 (3)	0.0700 (8)	
H12A	0.2246	−0.0169	0.1687	0.105*	
H12B	0.0369	−0.0287	0.2019	0.105*	
H12C	0.1978	−0.0045	0.3094	0.105*	

### Atomic displacement parameters (Å^2^)

**Table:**

	*U*^11^	*U*^22^	*U*^33^	*U*^12^	*U*^13^	*U*^23^
S	0.0374 (3)	0.0352 (3)	0.0829 (5)	−0.0002 (2)	−0.0066 (3)	−0.0011 (3)
N1	0.0309 (9)	0.0361 (9)	0.0641 (12)	0.0015 (7)	−0.0027 (8)	0.0017 (9)
N2	0.0339 (9)	0.0374 (10)	0.0598 (12)	−0.0031 (7)	−0.0003 (8)	0.0001 (9)
N3	0.0273 (8)	0.0326 (8)	0.0443 (10)	0.0001 (7)	0.0000 (7)	0.0000 (7)
N4	0.0305 (9)	0.0416 (10)	0.0507 (11)	0.0016 (8)	−0.0031 (8)	0.0013 (8)
C1	0.0304 (10)	0.0400 (11)	0.0444 (12)	0.0025 (8)	0.0028 (9)	0.0000 (9)
C2	0.0333 (11)	0.0368 (11)	0.0449 (12)	−0.0042 (8)	0.0042 (9)	−0.0016 (9)
C3	0.0468 (12)	0.0380 (12)	0.0586 (14)	−0.0010 (10)	0.0044 (11)	0.0004 (11)
C4	0.0662 (16)	0.0398 (13)	0.0762 (18)	−0.0139 (11)	−0.0015 (14)	0.0007 (12)
C5	0.0336 (11)	0.0393 (11)	0.0568 (14)	0.0026 (9)	0.0011 (10)	0.0017 (10)
C6	0.0297 (10)	0.0505 (12)	0.0423 (12)	0.0011 (9)	0.0042 (9)	0.0005 (10)
C7	0.0349 (11)	0.0594 (15)	0.0493 (14)	−0.0029 (10)	0.0042 (10)	−0.0021 (11)
C8	0.0336 (12)	0.090 (2)	0.0625 (17)	−0.0080 (13)	0.0014 (12)	−0.0046 (15)
C9	0.0353 (13)	0.104 (2)	0.0637 (17)	0.0157 (15)	−0.0003 (12)	0.0089 (16)
C10	0.0513 (14)	0.0725 (17)	0.0615 (16)	0.0261 (13)	0.0086 (13)	0.0125 (14)
C11	0.0413 (12)	0.0545 (14)	0.0538 (14)	0.0100 (10)	0.0069 (11)	0.0033 (11)
C12	0.0550 (15)	0.0620 (17)	0.089 (2)	−0.0124 (13)	0.0029 (14)	−0.0045 (15)

### Geometric parameters (Å, °)

**Table:**

S—C1	1.679 (2)	C5—C6	1.469 (3)
N1—C1	1.338 (2)	C5—H5	0.9300
N1—N2	1.373 (2)	C6—C11	1.393 (3)
N1—H1N	0.9314	C6—C7	1.399 (3)
N2—C2	1.296 (2)	C7—C8	1.398 (3)
N3—C1	1.378 (2)	C7—C12	1.497 (3)
N3—C2	1.384 (2)	C8—C9	1.369 (4)
N3—N4	1.395 (2)	C8—H8	0.9300
N4—C5	1.255 (2)	C9—C10	1.376 (4)
C2—C3	1.479 (3)	C9—H9	0.9300
C3—C4	1.522 (3)	C10—C11	1.382 (3)
C3—H3A	0.9700	C10—H10	0.9300
C3—H3B	0.9700	C11—H11	0.9300
C4—H4A	0.9600	C12—H12A	0.9600
C4—H4B	0.9600	C12—H12B	0.9600
C4—H4C	0.9600	C12—H12C	0.9600
			
C1—N1—N2	114.48 (16)	N4—C5—H5	120.2
C1—N1—H1N	122.2	C6—C5—H5	120.2
N2—N1—H1N	123.2	C11—C6—C7	120.14 (19)
C2—N2—N1	104.15 (15)	C11—C6—C5	119.32 (19)
C1—N3—C2	108.74 (15)	C7—C6—C5	120.53 (19)
C1—N3—N4	132.83 (15)	C8—C7—C6	117.7 (2)
C2—N3—N4	118.30 (15)	C8—C7—C12	119.6 (2)
C5—N4—N3	119.38 (17)	C6—C7—C12	122.71 (19)
N1—C1—N3	102.21 (16)	C9—C8—C7	121.7 (2)
N1—C1—S	127.10 (15)	C9—C8—H8	119.2
N3—C1—S	130.66 (14)	C7—C8—H8	119.2
N2—C2—N3	110.40 (17)	C8—C9—C10	120.5 (2)
N2—C2—C3	126.69 (18)	C8—C9—H9	119.8
N3—C2—C3	122.91 (17)	C10—C9—H9	119.8
C2—C3—C4	112.41 (18)	C9—C10—C11	119.3 (2)
C2—C3—H3A	109.1	C9—C10—H10	120.3
C4—C3—H3A	109.1	C11—C10—H10	120.3
C2—C3—H3B	109.1	C10—C11—C6	120.7 (2)
C4—C3—H3B	109.1	C10—C11—H11	119.6
H3A—C3—H3B	107.9	C6—C11—H11	119.6
C3—C4—H4A	109.5	C7—C12—H12A	109.5
C3—C4—H4B	109.5	C7—C12—H12B	109.5
H4A—C4—H4B	109.5	H12A—C12—H12B	109.5
C3—C4—H4C	109.5	C7—C12—H12C	109.5
H4A—C4—H4C	109.5	H12A—C12—H12C	109.5
H4B—C4—H4C	109.5	H12B—C12—H12C	109.5
N4—C5—C6	119.54 (19)		

### Hydrogen-bond geometry (Å, °)

**Table:**

*D*—H···*A*	*D*—H	H···*A*	*D*···*A*	*D*—H···*A*
N1—H1N···S^i^	0.93	2.37	3.2899 (19)	169
C5—H5···S	0.93	2.54	3.239 (2)	132

Symmetry codes: (i) −*x*+2, −*y*, −*z*+1.
